# Impact of Y181C and/or H221Y mutation patterns of HIV-1 reverse transcriptase on phenotypic resistance to available non-nucleoside and nucleoside inhibitors in China

**DOI:** 10.1186/1471-2334-14-237

**Published:** 2014-05-05

**Authors:** Wei Guo, Hanping Li, Daomin Zhuang, Liyan Jiao, Siyang Liu, Lin Li, Yongjian Liu, Tao Gui, Lei Jia, Jingyun Li

**Affiliations:** 1 Department of AIDS Research, State Key Laboratory of Pathogen and Biosecurity, Beijing Institute of Microbiology and Epidemiology, 20 Dongda Street, Fengtai District, Beijing 100071, China

**Keywords:** Nucleoside reverse transcriptase inhibitors (NRTIs), Non-nucleoside reverse transcriptase inhibitors (NNRTIs), HIV-1 resistance mutation, 50% inhibitory drug concentration

## Abstract

**Background:**

The aim of this study was to investigate the role of K101Q, Y181C and H221Y emerging in HIV-1 reverse transcriptase with different mutations patterns in phenotypic susceptibility to currently available NNRTIs (nevirapine NVP, efavirenz EFV) and NRTIs (zidovudine AZT, lamivudine 3TC, stavudine d4T) in China.

**Methods:**

Phenotype testing of currently available NNRTIs (NVP, EFV) and NRTIs (AZT, 3TC, d4T) was performed on TZM-b1 cells using recombined virus strains. P ≤ 0.05 was defined significant considering the change of 50% inhibitory drug concentration (IC_50_) compared with the reference, while P ≤ 0.01 was considered to be statistically significant considering multiple comparisons.

**Results:**

Triple-mutation K101Q/Y181C/H221Y and double-mutation K101Q/Y181C resulted in significant increase in NVP resistance (1253.9-fold and 986.4-fold), while only K101Q/Y181C/H221Y brought a 5.00-fold significant increase in EFV resistance. Remarkably, K101Q/H221Y was hypersusceptible to EFV (FC = 0.04), but was significantly resistant to the three NRTIs. Then, the interaction analysis suggested the interaction was not significant to NVP (F = 0.77, P = 0.4061) but significant to EFV and other three NRTIs.

**Conclusion:**

Copresence of mutations reported to be associated with NNRTIs confers significant increase to NVP resistance. Interestingly, some may increase the susceptibility to EFV. Certainly, the double mutation (K101Q/H221Y) also changes the susceptibility of viruses to NRTIs. Interaction between two different sites makes resistance more complex.

## Background

The reverse transcriptase (RT) of human immunodeficiency virus type 1 (HIV-1) is a multifunctional enzyme, possessing RNA-dependent DNA polymerase (RDDP) activity, DNA-dependent DNA polymerase (DDDP) activity and RNase H activity [1,2]. RT is an essential enzyme for the HIV-1 life-cycle. So it is the target for antiviral drugs in HIV-1 antiviral therapy [2-5]. There are two classes of HIV-1 RT inhibitors approved for the treatment of HIV-1 infection: nucleoside reverse transcriptase inhibitors (NRTIs) and non-nucleoside reverse transcriptase inhibitors (NNRTIs). Highly active antiretroviral therapy (HAART) generally comprises three antiretroviral drugs, usually two NRTIs and either PR inhibitors or a NNRTI drug [6]. So far, eight NRTIs and four NNRTIs have been used as parts of HAART. Drug pressure is responsible for the dramatic increase of such an intrinsic variability, which ends up with the final development of mutations, especially in the *pol* region encoding both RT and protease (PR) enzymes [7]. Constant HAART selects HIV-1 resistant virus which may have accumulated resistances to all the available drugs. Since the surveillance of HIV-1 drug resistance was approved in 2004 in China, a mass of data about the prevalence rate of HIV-1 drug resistance virus, influencing factors and the effect on antiviral therapy have been acquired. With the extending of the time of HAART and the improvement of the sensitivity of HIV-1 drug resistance testing, novel potential resistance associated mutations are being identified. Noteworthy, the prominent role of novel mutations in contributing to HIV-1 drug resistance, the interaction between different mutations and the change of replication capacity of HIV-1 resistant strains remain unclear. Some articles have predicted that as additional or secondary mutations, novel mutations combined with those currently known are involved in NNRTIs resistance by directly increasing resistant level of RT inhibitors or compensating the loss of replication capacity [8]. Therefore, they lead to antiretroviral therapy failure [9].

First-generation NNRTIs have a low genetic barrier for resistance. Only a single-nucleotide change can result in high-level resistance with little impact on the replication [10]. Moreover, mutations are stable and hardly reverse to wild types in absence of drug pressure [11-14]. Analyses of HIV-1 RT crystallographic indicated that the polymerase activity can be significantly influenced by conformational changes that occur in an allosteric site known as NNRTI binding pocket (NNRTI-BP) [7,15]. Amino acids substitutions located at NNRTI-BP induce NNRTI-resistance (L100, K101, K103, E138, V179, Y181 and Y188) [16]. Furthermore, many studies were focused on the probalble mechanism of resistance mutations and they were assisted by experiments [4,17,18]. However, the common NNRTI mutations were K103N and Y181C whose roles in NNRTI-resistance have been clarified [14,19,20]. Recent studies have confirmed novel mutations are positively associated with NNRTIs treatment [8,21-23]. Ceccherini-Silberstein reported that novel mutations may actively participate in the NNRTIs resistance and the development of NNRTI resistance may be more complex (≥3 NNRTI resistance mutations) than the first-generation NNRTIs resistance [9]. However, viral resistance depends not only on the accumulation of an increasing number of mutations over time, but also on the specific combination of mutations [21]. H221Y, which had been believed to emerge in NRTI-treatment patients and considered to be polymorphism [8,9,21], proved to be a novel mutation correlated with NNRTI-resistance in 2003. However, it had been certified that the frequency of H221Y significantly increased in NNRTI-treatment failing patients compared with drug-naïve and NRTI-treated NNRTI-naïve patients [9,21,24]. Moreover, H221Y was strongly associated with the use of NVP and showed positive interactions with Y181C [9,25]. It was demonstrated that K101Q with H221Y as an unreported HIV-1 RT mutation pattern was associated with phenotypic resistance to the NNRTI class [21]. Other studies showed the mutations conferring resistance to one class could change the susceptibility of viruses to the others [26].

Here, we are focused on defining the role of K101Q, Y181C, H221Y emerging in different patterns. To investigate whether these mutations may confer a decreased phenotypic susceptibility to currently available NNRTIs (nevirapine NVP, efavirenz EFV) and NRTIs (AZT, lamivudine 3TC, stavudine d4T) in China. At last, we analyze the potential interaction between sits 181 and 221 in the background of K101Q.

## Methods

### Patients and samples

We traced six patients for 47–58 months in Henan Province failing two NRTIs plus one NNRTI [zidovudine (AZT) plus didanosine (ddI) plus nevirapine (NVP)]. They were infected HIV-1 subtype B by blood donation. Patients complied with treatment regimens well. We had followed up with interval for approximately six months (ten times) since the very start of therapy. Every time, we collected 10 ml anticoagulated whole blood samples, separated them by centrifugation to obtain blood plasma and peripheral blood mononuclear cells (PBMCs) and then stored them at -80°C.

### Clonal sequencing of HIV-1 in plasma and PBMCs

Clonal sequencing approach was adopted in this study [27]. RNA and DNA were extracted from plasma and PBMCs respectively according to the manufacturer’s instructions of QIAamp as the template for a nested PCR [25,28]. PCR products were independently cloned, and a single clone was sequenced. Thus, each sequence reflected the genotype of an independent viral genome. The nucleotide sequence of a 2.1 kb segment of the HIV-1 genome included the entire protease and RT coding region. We analyzed these sequences at each follow-up time.

### Construction of recombined virus

Amplification of viral genome was performed using a nested PCR procedure, and patient-derived HIV-1 RT fragment carrying K101Q/Y181C/H221 replaced the partner sequence (2843 nt-3485 nt, 643 bp) in pNL4-3 *pol* to construct the first clone as previously described [29]. There were few other mutations reported to be associated with resistance in Stanford drug resistance database. Then, site-directed mutagenesis was carried out to obtain another three HIV-1 clones separately harboring K101Q/Y181C, K101Q/H221Y and K101Q. Recombined pNL4-3 plasmids and wild-type pNL4-3 as the control were transfected into HEK293T cells using Lipofectamine 2000 following the manufacturer’s instructions and harvested mutant or wild-type viruses transfection supernatant at the 48^th^ hour. Then, transfection supernatant infected MT-2 cells and viral cultures were grown in 4 to 6 days. Supernatants were stored at -80°C and sequenced to confirm the presence of the desired mutations. Although we constructed the single mutation (H221Y) virus as mentioned above to clarify the contribution of H221Y to resistance, the virus was low virus titer. So we did not obtain a reliable result of single H221Y.

### Phenotypic drug susceptibility assays

The study tested the susceptibility of viruses to currently available RT inhibitors (NVP, EFV, AZT, 3TC and d4T) in China with recombined viruses. They were performed in TZM-b1 cells as previously described [29]. In brief, drugs at variable concentrations were added to TZM-bl cells (10^4^cells/well) in 96-well plates growing in 100ul Dulbecco’s minimal essential medium (DMEM) (Gibco) supplemented with 10% fetal bovine serum (Gibco), 1% penicillin-streptomycin [30]. Immediately after drugs addition, cells were infected with wild-type or mutant viruses normalized by TCID_50_. Forty-eight hours after the TZM-b1 got infected, with the condition of 37°C and 5% CO_2_, relative luminescence units (RLU)/well were measured by a luminometer (Wallik 1420; Perkin Elmer) according to Bright-glo^TM^ Luciferase assay system (Promage E2650) instructions. All the experiments were performed at least in duplicate on three different days. The IC50 was calculated using the GraphPad Prism program.

### Statistical analysis

Multiple comparisons statistical method was used to assess the significance of differences in IC_50_ values between any two viruses, and Kruskal-Wallis method was used to correct the testing. P ≤ 0.05 was defined significant considering the change of 50% inhibitory drug concentration (IC_50_) compared with the reference, while P ≤ 0.01 was considered to be statistically significant considering multiple comparisons.

### Ethical consideration

The study was approved by the Ethical Board of the Beijing Institute of Microbiology and Epidemiology in January 2009. All the patients were compliance with the first antiviral therapy program in China and were selected with informed consent. Data were managed anonymously.

## Results

H221Y, a novel NNRTI-resistance mutation relevant to NVP, emerged in all the six patients. There were 204 sequences of Patient 1 blood plasma and 160 of PBMCs. Combination of H221Y and Y181C was detected in the 10^th^ month of antiviral therapy with the frequency more than 30% in quasispecies. From the 22^nd^ month on, the frequency of H221Y/Y181C in quasispecies was 100% in plasma. However, not until the 28^th^ month did the frequency of H221Y/Y181C in quasispecies become 100% in PBMCs. Remarkably, K101Q was observed along with the double mutations at the early time of antiviral therapy.

Apart from the above, we also observed more mutations in plasma than in PBMCs at each interview time. In other words, some mutations which emerged in plasma were not found in PBMCs. With the antiviral therapy going, mutations in PBMCs and plasma would be consilient. However, certain mutations were momentary and were absent in the last mutations patterns. All the mutations present in the last patterns were nearly 100% in both PBMCs and plasma.

### Recombined viruses

We obtained one reference virus pNL4-3_*WT*_ and 4 recombined HIV-1 viruses separately harboring K101Q/Y181C/H221Y, K101Q/Y181C, K101Q/H221Y and K101Q. By analyzing sequences, we confirmed that the desired mutations did exist. Then, we calculated IC_50_ of 5 drugs (NVP, EFV, AZT, 3TC and d4T) by making dose-effect relationship using the GraphPad Prism program. Moreover, the fold changes (FC) of IC_50_ were calculated in Table 1.

**Table 1:** **The IC**_**50 **_**of 5 viruses and FC contrasted with pNL4-3**_*W* ***T***_

**Virus**	**IC**_**50**_^**a**^ **± SD (nM) (FC**^**b**^**)**				
	**NVP**	**EFV**	**AZT**	**3TC**	**d4T**
WT_pNL4–3_	74.49 ± 9.24 (1.0)	13.66 ± 7.74 (1.0)	21.42 ± 16.84 (1.0)	44.60 ± 31.99 (1.0)	275.20 ± 118.69(1.0)
K101Q/Y181C/H221Y	93400.00 ± 32831.88*(1253.9)	68.33 ± 12.95*(5.0)	40.90 ± 13.55 (1.9)	102.38 ± 30.42 (2.3)	727.55 ± 157.47 (1.9)
K101Q/Y181C	73470.00 ± 20855.22*(986.4)	40.46 ± 15.70 (3.0)	10.26 ± 4.34 (0.5)	79.17 ± 3.56 (1.2)	373.33 ± 214.30 (1.4)
K101Q/H221Y	294.63 ± 80.62 (4.0)	0.54 ± 0.32*(0.04)	222.10 ± 49.35*(10.4)	496.8 ± 115.53*(11.1)	5715.00 ± 610.50*(20.8)
K101Q	56.63 ± 11.36 (0.8)	5.17 ± 1.44 (0.4)	52.34 ± 11.93 (2.4)	82.58 ± 62.18 (1.9)	322.90 ± 119.67 (1.2)

a, the 50% inhibitory concentration. b, calculated by dividing the IC50 of each mutant virus by the IC50 for the wild-type. The mean ± SD of three independent experiments. FC only showed the mean of three independent experiments. * IC50 was significantly change compared with pNL4-3_*WT*_, *P* < 0.05.

### The changes of NVP and EFV susceptibility induced by association mutation

**Figure 1:**
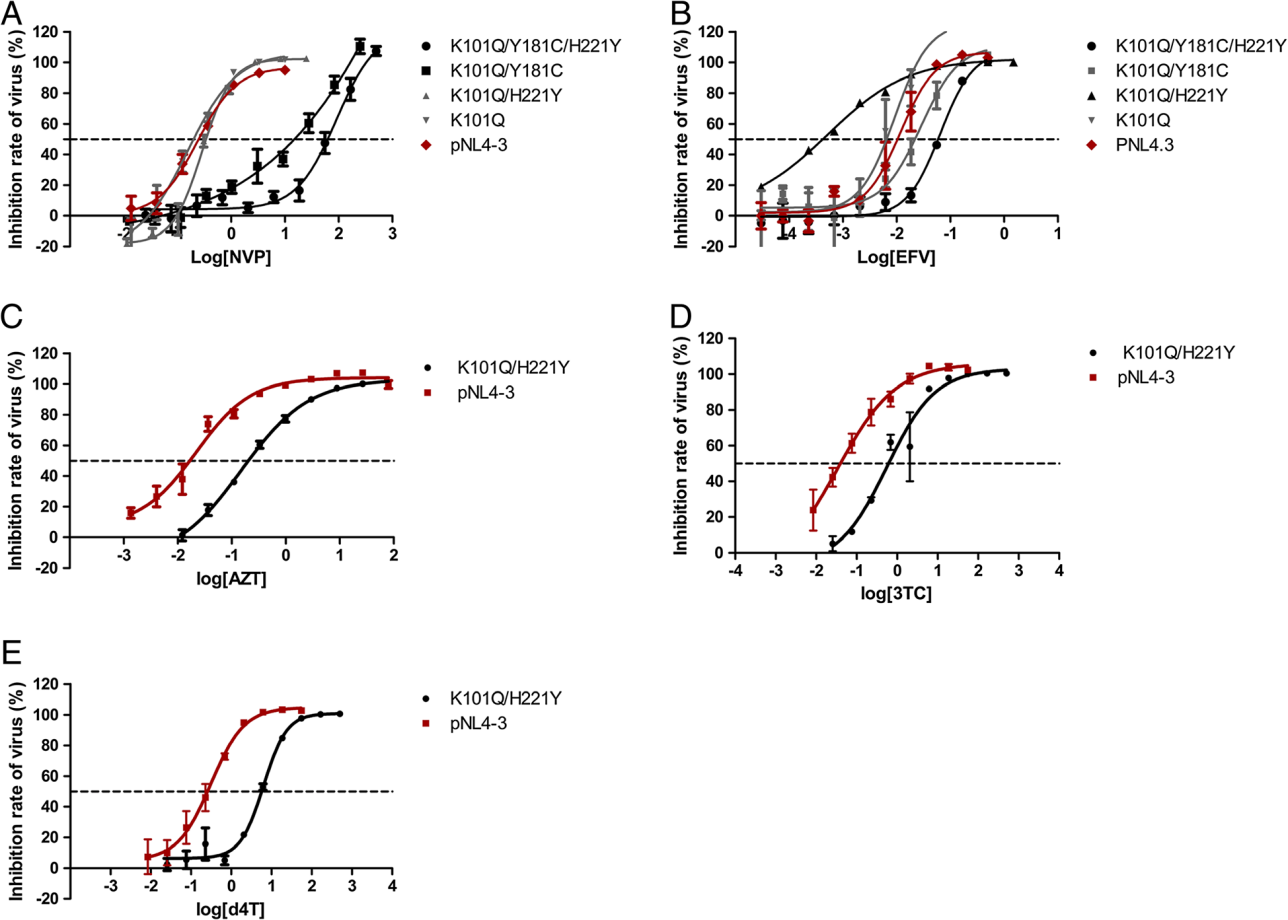
**Curve of the dose-effect relationship of different drugs.** x axes indicate log of drug concentrations (uM), y axes indicate the inhibitor rate of virus (%). Red curve is the dose-effect relationship of control pNL4-3, dotted line is y = 50%. **(A)** is the dose-effect relationship of 5 viruses (K101Q/Y181C/H221Y, K101Q/Y181C, K101Q/H221Y, K101Q and pNL4-3) in NVP. **(B)** is the dose-effect relationship of 5 viruses (K101Q/Y181C/H221Y, K101Q/Y181C, K101Q/H221Y, K101Q and pNL4-3) in EFV. **(C)**, **(D)**, **(E)** is the dose-effect relationship of K101Q/H221Y and pNL4-3 in AZT, 3TC and d4T respectively.

To assess the direct contribution of mutation patterns to NVP and EFV, we analyzed the IC_50_ data of NVP and EFV in Table 1 with multiple testing statistical methods. Kruskal-Wallis method was used to correct multiple tests with the false-discovery rate of 0.05. We observed viruses containing K101Q/Y181C/H221Y result in a 5.00-fold significant increase in EFV resistance and a 1253.9-fold increase in NVP resistance. The copresence of K101Q and Y181C in viruses resulted in a little increase in EFV resistance, but a 986.4-fold increase in NVP resistance. Interestingly, K101Q plus H221Y contributed to a significant 25.00-fold decrease in EFV resistance while a 4.0-fold increase in NVP resistance. K101Q caused 2.63-fold and 1.32-fold change of IC_50_ respectively (Figure 1A-B).

### The effect of association mutation patterns on NRTIs

A further step of this study was to investigate whether the susceptibility to NRTIs was also altered by these mutations (Figure 1C-E). Table 1 shows viruses carrying K101Q slightly altered resistance to AZT, 3TC and d4T, and the same as K101Q/Y181C viruses. Viruses harboring K101Q/H221Y displayed significantly resistance to AZT, 3TC and d4T (10.37-fold, 11.14-fold and 20.77-fold). However, the viruses, Y181C along with K101Q and H221Y, presented a little IC_50_ increase of the three NRTIs.

### The interaction between Y181C and H221Y at the background of K101Q

Regarding K101Q as the background, we analyzed the interaction between Y181C and H221Y of five drugs respectively. We found the interaction between sites 181 and 221 was not statistically significant (F = 0.77, P = 0.4061) in the presence of NVP, but the interaction was statistically significant with EFV (F = 12.80, P = 0.0072). Comparing K101Q/Y181C/H221Y with K101Q in the presence of EFV, we found the contribution of Y181C plus H221Y to resistance was 13.22-fold which was higher than the sum of the folds of K101Q/Y181C versus K101Q and K101Q/H221Y versus K101Q. We guessed the interaction between Y181C and H221Y was synergetic. We also analyzed the interaction between sites 181 and 221 in NRTIs at the background of K101Q. Results showed the interaction was significant in AZT, 3TC and d4T (Table 2). Table 1 displayed the FC in three NRTIs as the following: K101Q/H221Y>K101Q/Y181C/H221Y>K101Q/Y181C. We speculated Y181C had significantly decreased the H221Y resistance to the three NRTIs at the background of K101Q.

**Table 2:** Interaction between Y181C and H221Y at the background of K101Q (alpha = 0.05)

**Drugs**	181*221^**a**^	
	**F**	**P**
NVP	0.77	0.4061
EFV	12.80	0.0072
AZT	20.87	0.0018
3TC	19.98	0.0021
d4T	144.56	<.0001

a, interaction between 181 and 221; the *P* was the probabilities of no significant interaction between 181 and 221. *, the interaction between sits 181 and 221.

## Discussion

Compared with the genotypic drug resistance test, the phenotypic drug resistance assay is a test of replication capability at presence of drug in vitro. Most of the phenotypic susceptibility tests are based on constructing fragments from virus infected patients to the backbone of subtype B. Although it is restricted to the backbone of subtype B, it has been considered as golden standard to evaluate phenotypic susceptibility [31]. In many studies, parts of RT genes were inserted into pNL4-3 clone to create recombined HIV-1 viruses and then to develop associated susceptibility tests. Although our HIV-1 strains belonged to B’ subtype, all the mutations were defined as the amino acids that differed from the HIV-1 consensus B sequence.

In the pre-study, we found the viruses carrying H221Y were commonly combined with Y181C, the same result as the one in the reference papers [19,25]. At the same time, the double mutations were observed along with other NNRTIs including K101E, K101Q, V179D, V179E, K103N, and the viruses were predominant in quasispecies of 6 HIV-1 infected patients in a drug resistance surveillance cohort. Previous studies demonstrated K101Q was not correlated with any NNRTI resistance mutations, but was the prerequisite to the presence of K103N [9,27]. However, we observed K101Q emerge along with Y181C and H221Y, but not with K103N. The observed patterns of correlated mutations may be affected by pharmacological pressure and imposed by the drug regimens that were used in different cohorts [9].

Long NNRTIs exposure may trigger the accumulation of additional mutations, leading to even higher levels of drug resistance. Here, we evaluated the contribution of K101Q plus Y181C plus H221Y to resistance of NNRTIs (NVP, EFV). The result showed K101Q/Y181C/H221Y viruses led to a 1253.9-fold great increase in NVP resistance and a 5.00-fold significant increase in EFV resistance. The copresence of K101Q and Y181C in viruses resulted in a little increase in EFV resistance, but a 986.4-fold increase in NVP resistance. However, K101Q was interpreted as a relatively non-polymorphic mutation that occurred slightly more commonly among patients receiving NNRTIs. It was reported that single K101Q induced a 3.2-fold NVP resistance and a 5.6-fold EFV resistance and that Y181C conferred 100-fold NVP resistance and 1.1-fold EFV resistance at the pNL4-3 background [19]. Moreover, Y181C was known to confer high level resistance to NVP [18]. Hypersusceptibility should be identified under two conditions: One is that IC_50_ of the test viruses were significantly less than wild type. The other is that fold-change values was less than 0.4 compared with wild-type control virus run in parallel [32,33]. K101Q hardly changed susceptibility of NVP and EFV, with FC value of 0.8-fold and 0.4-fold compared with wild-type respectively. However, it is interpreted in Stanford database that H221Y does not decrease susceptibility by itself but may contribute to the decrease of NNRTI susceptibility in combination with other NNRTI-resistance mutations. In this study, we observed copresence of the two secondary mutants (K101Q and H221Y) show hypersusceptibility to EFV with a mean IC_50_ value of 0.54 ± 0.32nM (FC = 0.04, P<0.05), but only a 4.0-fold increase in NVP resistance. How these mutations cause NNRTI resistance is not clear. It is conceivable that more mutations or associated mutations than currently known are involved in the development of drug resistance and lead to therapeutic failure [5]. In particular, novel mutations participate in the NNRTI resistance may be more complex (≥3 NNRTI resistance mutations) than the first-generation NNRTIs resistance [9].

We also investigated whether these mutations conferred NRTI resistance. K101Q/H221Y double mutation showed significantly increase in AZT, 3TC, d4T resistance. It is worth noting that triple-mutation K101Q/Y181C/H221Y only shows a less extensive increase. The situation is made complicated by the fact that resistance mutations do not accumulate independently within each other. Instead, they disappear and occur in time order along the pathway of resistance evolution, leading to distinct mutational complexes or clusters [34]. The more mutations are combined together, the more complex their mechanisms are.

We evaluated the interaction between Y181C and H221Y considering K101Q as the background. Result of statistical analyses showed the interaction between sites 181 and 221 in NVP was not statistically significant (F = 0.77, P = 0.4061). In EFV, H221Y increased the susceptibility while Y181C increased resistance. However, Y181C significantly reversed the K101Q/H221Y phenotypic susceptibility to EFV. Then, as for NRTIs, Y181C significantly decreased the H221Y resistance. Mutational pathways may interpret the complexity at a certain extent. Regrettably, we have not observed which one first emerges during mutational pathways, Y181C or H221Y. So far, some researchers have reported the molecular mechanism between the Y181C and other mutations [4]. Although Ceccherini-Silberstein reported novel mutations cluster (L74V and H221Y) frequently appears with Y181C and share with it the ability to increase NNRTI resistance [9], but the idiographic impact and molecular mechanism were unclear.

## Conclusions

In summary, some copresence of the mutations reported to be associated with NNRTIs in our study confer significant increase of NVP resistance. Interestingly, some may increase the susceptibility of EFV. Certainly, the double mutation (K101Q/H221Y) also changes the susceptibility of viruses to NRTIs. Interaction between different sites makes resistance more complex.

Our data in this study are based on the recombined subtype B’ viruses and pNL4-3 wild-type. Viral evolution pathways toward drug resistance may proceed through distinct steps and at different rates among different HIV-1 subtypes [10]. To assess the prevalence of novel cluster mutations in other non-B’ subtypes and to test the role of them to resistance is necessary. Further analyses on the structure of novel RT mutation clusters will provide physic-theory of the resistance. However, additional studies in vitro will be necessary to distinguish and highlight their mechanisms of action better.

## Abbreviations

HIV-1: Human immunodeficiency virus type 1; HAART: Highly active antiretroviral therapy; NRTIs: Nucleoside reverse transcriptase inhibitors; NNRTIs: Non-nucleoside reverse transcriptase inhibitors; IC_50_: 50% inhibitory concentration; RT: HIV-1 reverse transcriptase; NVP: Nevirapine; EFV: Efavirenz; AZT: Zidovudine; 3TC: Lamivudine; d4T: Stavudine.

## Competing interests

The authors declare that they have no competing interests.

## Authors’ contributions

Conceived and designed the experiments: JL, WG, HL, DZ. Performed the experiments: WG, DZ, SL, LJ. Analyzed the data: WG, LJ, TG. Wrote the paper: WG, LL, YL. All authors read and approved the final manuscript.

## Pre-publication history

The pre-publication history for this paper can be accessed here:

http://www.biomedcentral.com/1471-2334/14/237/prepub
